# Treatment outcomes in acute invasive fungal rhinosinusitis extending to the extrasinonasal area

**DOI:** 10.1038/s41598-020-60719-7

**Published:** 2020-02-28

**Authors:** Sung Hoon Nam, Yoo-Sam Chung, Young Jun Choi, Jeong Hyun Lee, Ji Heui Kim

**Affiliations:** 1 Department of Otorhinolaryngology-Head and Neck Surgery, Asan Medical Center, University of Ulsan College of Medicine, Seoul, Republic of Korea; 2 Department of Radiology and Research Institute of Radiology, Asan Medical Center, University of Ulsan College of Medicine, Seoul, Korea

**Keywords:** Fungal infection, Magnetic resonance imaging

## Abstract

Acute invasive fungal rhinosinusitis (AIFRS) can spread beyond the sinonasal cavity. It is necessary to analyze the association between the specific site involved in the extrasinonasal area and the survival rate to predict patient prognosis. We investigated 50 patients who had extrasinonasal lesions on preoperative gadolinium (Gd)-enhanced magnetic resonance imaging (MRI) scan and underwent wide surgical resection of AIFRS. The specific sites with loss of contrast enhancement (LoCE) on Gd-enhanced MRI were analyzed for AIFRS-specific survival rate. The most common underlying disease was diabetes mellitus followed by hematological malignancy. The most common symptoms were headache and facial pain. Seven patients (14.0%) expired because of AIFRS progression. Poor prognosis was independently associated with LoCE at the skull base on preoperative MRI (HR = 35.846, *P* = 0.004). In patients with AIFRS extending to the extrasinonasal area, LoCE at the skull base was an independent poor prognostic factor.

## Introduction

Acute invasive fungal rhinosinusitis (AIFRS) is a rare but fatal disease, particularly in immune-compromised patients, including those with hematological malignancies, poorly controlled diabetes mellitus (DM), and those who received immunosuppressive treatment following organ transplantation or chemotherapy for solid organ malignancies^1–3^. Surgical debridement, systemic antifungal therapy, and prompt correction of the underlying systemic disease are necessary for treatment of AIFRS^4,5^.

A recent systematic review of AIFRS reported a mortality rate of 49.7% that advanced age and intracranial involvement were independent poor prognostic factors, and that DM and surgical resection were good prognostic factors^6^. Surgical resection of necrotic tissue is an important treatment modality for AIFRS; therefore, the extent of AIFRS may be a significant prognostic factor. Recently, it has been shown that information from gadolinium (Gd)-enhanced magnetic resonance imaging (MRI) may be helpful in predicting prognosis^7^. Loss of contrast enhancement (LoCE) on MRI reveals tissue infarction secondary to vessel invasion by a fungus^8–10^, which cannot be infiltrated by antifungal agents and requires surgical resection. However, the surgeon may be reluctant to remove the extrasinonasal lesion if there is a lesion on the site that is likely to cause serious postoperative complications, including cerebrospinal fluid (CSF) leakage and large vessel injury. Therefore, analyzing the relationship between the specific site of involvement in the extrasinonasal area and the survival rate is important in predicting patient prognosis. To the best of our knowledge, there are no previous reports on the relationship between specific sites with LoCE on Gd-enhanced MRI and prognosis of AIFRS. In the present study, we aimed to analyze the clinical features of patients with AIFRS extending to the extrasinonasal area and to identify prognostic factors for AIFRS-specific survival rate in terms of specific sites involved.

## Results

### Headache and facial pain are the most common symptoms in AFIRS patients

Table 1 summarizes the patients’ clinical characteristics. Thirty male (60.0%) and twenty (40.0%) female patients were included. Their median age was 63.5 years (range, 12–84 years), and 32 (64.0%) patients were ≥60 years old. The median follow-up period was 9.2 months (range, 0.3–110.5 months). Twenty-eight (56.0%) patients had DM, and 15 (30.0%) patients had hematological malignancies. Eight (16.0%) patients had two or more underlying diseases, whereas four elderly (8.0%) patients had no underlying disease. The most common symptom was headache in 21 (42.0%) patients, followed by facial pain in 17 (34.0%) patients. Neutropenia (absolute neutrophil count ≤500/mm^3^), severe lymphocytopenia (lymphocyte count ≤100 cells/mm^3^), and monocytopenia (monocyte count ≤10 cells/mm^3^) at the time of diagnosis^5^ were identified in six (12.0%), eight (16.0%), and four (8.0%) patients, respectively. Seven (14.0%) patients had evidence of pulmonary fungal infection on chest CT at the time of AIFRS diagnosis.

**Table 1:** Underlying conditions and presenting symptoms of 50 patients with acute invasive fungal rhinosinusitis.

	No. of patients (%)
Underlying conditions	
Diabetes mellitus	28 (56.0)
Hematological malignancy	15 (30.0)
Solid organ transplantation	6 (12.0)
Solid organ malignancy with chemotherapy	5 (10.0)
Autoimmune neutropenia	1 (2.0)
Retuximab for refractory Wegener’s granulomatosis	1 (2.0)
Underlying conditions (≥1)	8 (16.0)
None	4 (8.0)
History of steroid use (≤1 month)	17 (34.0)
Presenting symptom	
Headache	21 (42.0)
Facial pain	17 (34.0)
Rhinorrhea	14 (28.0)
Nasal obstruction	13 (26.0)
Facial swelling	10 (20.0)
Ophthalmoplegia	8 (16.0)
Orbital swelling	7 (14.0)
Fever	9 (18.0)
Visual disturbance	6 (12.0)
Facial palsy	4 (8.0)
Facial paresthesia	4 (8.0)
Diplopia	2 (4.0)

All patients had definite invasion of fungus to mucosa, bone, vessel, and/or adjacent tissue on histopathological analysis. Thirty-three (66.0%) patients were mucormycosis, 16 (32.0%) patients were aspergillosis, and one patient’s infectious organism was not clarified. However, only 21 (42.0%) patients were positive in fungal culture. Thirty-four (68.0%) patients underwent preoperative empirical antifungal medication. Twenty-three patients with mucormycosis, and the patient infected with unknown species were treated with amphotericin B/liposomal amphotericin B-based therapy. The 16 patients with aspergillosis were treated with voriconazole. The median duration of antifungal treatment was 98 days (range, 9–599 days).

A total of 31 (62.0%) patients underwent an endoscopic approach only, and 19 (38.0%) patients underwent either a sublabial approach or maxillectomy. The median number of operations was two (range, 1–5 times), and 26 (52.0%) patients underwent surgery more than once. There were no serious postoperative complications, such as CSF leakage or uncontrolled hemorrhage. Eight patients with invasive mucormycosis and a patient with invasive aspergillosis who had preoperative LoCE lesions in the orbit underwent orbital exenteration, and all of them were histopathologically confirmed to have fungal invasion of orbital contents. Of them, three patients expired because of progression of residual lesions at the orbital apex and body of the sphenoid bone, respectively. An intracranial fungal abscess was identified in six (12.0%) patients on preoperative MRI, and two of them expired because of progression of residual lesions at body of the sphenoid bone and temporal lobe.

These clinical factors were not significantly associated with AIFRS-specific survival rate (all *P >* 0.05), except for well-controlled DM (*P* = 0.038, Table 2).

**Table 2:** Relationship between clinicopathological variables and acute invasive fungal sinusitis-specific survival.

Variable	P-value^a^
Age ≥ 60 years	0.172
Underlying conditions	0.582
Diabetes mellitus	0.859
Well-controlled diabetes mellitus	0.038
Hematological remission at time of diagnosis	0.749
Steroid use ≤1 month	0.648
Neutropenia	0.374
Lymphopenia	0.981
Monocytopenia	0.460
Pulmonary fungal infection	0.944
Organism (*Mucor*/*Aspergillus*)	0.307
Positive in fungal culture	0.970
Pre-emptive antifungal treatment	0.605

^a^Log-rank test. N/A, not available.

### LoCE at the skull base was an independent poor prognostic factor in AIFRS patient

After discontinuing the antifungal therapy, patients who did not show any evidence of AIFRS progression by endoscopy and MRI and did not relapse were judged to have been cured. According to this criterion, of 50 patients, 37 (74.0%) were cured of AIFRS at last follow-up (range, 0.3–110.5 months). 6 (12.0%) patients had no remnant lesion on endoscopy and MRI but died from progression of underlying disease or other infections during the administration of the antifungal agent. Seven (14.0%) patients expired within 100 days because of progression of AIFRS to the brain, which led to meningitis, cerebral hemorrhage, and/or infarction and brain edema. The incomplete resection sites in these seven patients comprised the body and/or greater wing of the sphenoid bone, orbital apex, and adjacent large area of dura.

**Figure 1:**
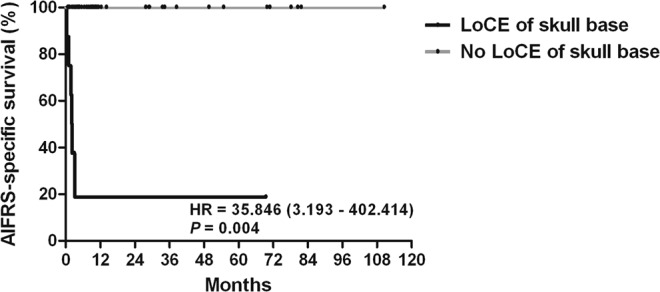
Kaplan–Meier survival curve for LoCE of the skull base. AIFRS, acute invasive fungal rhinosinusitis; HR, hazard ratio; LoCE, loss of contrast enhancement.

**Figure 2:**
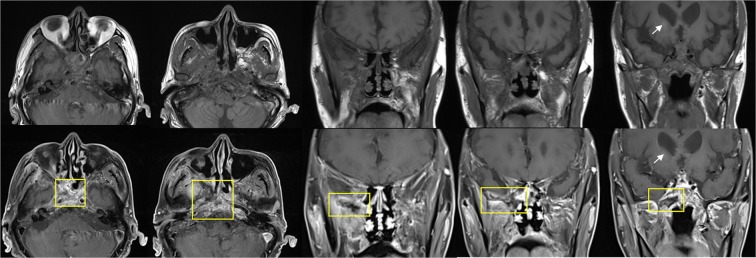
Preoperative MR images of a 69-year-old male patient who had extensive extrasinonasal acute invasive fungal sinusitis lesions and died of acute invasive fungal sinusitis progression. T1-weighted images (upper) and gadolinium-enhanced T1-weighted images (lower) show that right middle cranial fossa skull base, zygoma, maxillary sinus, pterygopalatine fossa, infratemporal fossa, and cavernous sinus were involved with mucormycosis. loss of contrast enhancement lesions reflecting a necrosis (yellow square) were observed in the right greater wing of sphenoid bone, sphenoid body, right pterygopalatine fossa, right infratemporal fossa, right pterygoid plate, right zygoma, and right maxillary sinus posterior wall. Soft tissue inflammation was identified in the right orbital apex, right cavernous sinus with internal carotid artery narrowing, both prevertebral space, right parapharyngeal space, and masticator space. Acute infarction in the right basal ganglia (white arrow) and enhancement of meninx were also found. Finally, he died of infarction of right anterior cerebral artery territory due to meningoencephalitis and skull base osteomyelitis.

When the relative risk of AIFRS-specific mortality according to the subsite of preoperative LoCE lesion was analyzed, the involvement of the orbit [hazard ratio (HR) =4.862, *P* = 0.038)], skull base (HR = 67.979, *P* = 001), cavernous sinus (HR = 9.873, *P* = 003), infratemporal fossa (HR = 11.238, *P* = 0.002), and meninges (HR = 11.238, *P* = 0.002) significantly increased the mortality rate in the univariate analysis (Table 3). In the multivariate analysis, only skull base with LoCE was an independent prognostic subsite [HR = 35.846; 95% confidence interval (CI), 3.193–402.414, *P* = 0.004, Table 4, Figs. 1 and 2].

**Table 3:** Univariate Cox proportional hazards regression model of the associations between acute invasive fungal sinusitis-specific survival and loss of contrast enhancement sites before surgery.

Site	No. of patients (%)	HR (95% CI)	*P*-value
Palate	7 (14.0)	2.394 (0.463–12.366)	0.297
Orbit	7 (14.0)	4.862 (1.087–21.738)	0.038
Skull base	7 (14.0)	67.979 (7.982–578.943)	0.001
Cavernous sinus	5 (10.0)	9.873 (2.160–45.121)	0.003
Pterygopalatine fossa	14 (28.0)	3.604 (0.806–16.122)	0.093
Infratemporal fossa	7 (14.0)	11.238 (2.480–50.934)	0.002
Meninges	7 (14.0)	11.238 (2.408–50.934)	0.002
Brain	6 (12.0)	3.915 (0.753–20.346)	0.105

HR, hazard ration; CI, confidence interval.

**Table 4:** Multivariate Cox proportional hazards regression model of the associations between acute invasive fungal sinusitis-specific survival and preoperative loss of contrast enhancement sites.

Site	HR (95% CI)	*P*-value
Orbit	2.274 (0.298–17.370)	0.429
Skull base	35.846 (3.193–402.414)	0.004
Cavernous sinus	5.232 (0.495–55.361)	0.169
Infratemporal fossa	1.771 (0.014–232.032)	0.818
Meninges	1.771 (0.014–232.032)	0.818

HR, hazard ratio; CI, confidence interval.

## Discussion

The treatment strategy for AIFRS involves extensive removal of necrotic lesions combined with antifungal therapy. AIFRS confined to the sinonasal cavity can be readily treated by endoscopic sinus surgery. However, when AIFRS extends beyond the sinonasal cavity, the surgeon has to predict the prognosis and determine the extent of surgical removal. In this study, the involvement of the skull base, particularly in the body and/or greater wing of the sphenoid bone and orbital apex, was identified as an independent poor prognostic factor in AIFRS extending to the extrasinonasal area.

The presenting symptoms of AIFRS are variable. A systematic review of AIFRS showed that the most common symptom was facial swelling (64.5%), followed by fever (62.9%), nasal congestion (52.2%), ophthalmoplegia (50.9%), facial pain (46.8%), and headache (46.3%)^6^. Conversely, a previous study reported that the most common symptoms were headache (61.1%), facial pain (55.6%), and ophthalmoplegia (33.3%)^11^. In another study, fever was the predominant presenting symptom, followed by facial pain, nasal obstruction, and rhinorrhea^12^. In the present study, facial pain, headache, and/or ophthalmoplegia were the most common symptoms, whereas nasal symptoms, including rhinorrhoea and nasal obstruction, occurred in only approximately 28% of patients. Moreover, fever was present in only nine (16.0%) patients, which was relatively low for a presenting symptom compared with those in other studies^6,12^. Fever may be more common in patients with hematological malignancy given that four out of five patients with fever had hematological malignancy. This study included a relatively small proportion of hematological malignancies (30.0%) with more than half being DM, whereas 39.0% and 90.1% of hematological malignancies were included in other studies^6,12^. Therefore, immunocompromised patients who complain of facial pain, headache, and/or ophthalmoplegia without other symptoms of sinusitis should be examined for the possibility of AIFRS.

The present study revealed that patients with well-controlled DM showed better AIFRS-specific survival as some previous studies, have shown that better prognosis was observed in patients with DM that can be rapidly managed^6,7,13^. However, advanced age and remission of hematological diseases at the time of AIFRS diagnosis were not significantly associated with AIFRS-specific survival rate in patients with AIFRS who underwent surgery. This result seems to be because the proportion of patients aged ≥60 years was relatively high (64.0%), and the proportion of patients with hematological diseases among those who died was relatively low (42.9%).

Although some studies have demonstrated that invasive mucormycosis showed higher mortality compared with invasive aspergillosis^14,15^, others have reported that the type of fungal organism was not associated with the survival rates of patients with AIFRS^6,7^. We also found that there was no significant difference between the survival rate of invasive mucormycosis and that of aspergillosis. A recent multicenter study reported that atypical fungi, including *Candida*, *Fusarium*, and *Alternaria*, which are resistant to amphotericin B and other antifungal agents, were associated with poor survival rate^16^. Considering the efficacy of antifungal agents, our results may be because these organisms have individual susceptibilities to amphotericin B and voriconazole. In addition, approximately 70% of patients with suspected AIFRS received empirical antifungal therapy before diagnostic and therapeutic surgery. Therefore, empirical antifungal treatment may not affect the AIFRS-specific survival rate.

Preoperative and postoperative extrasinonasal LoCE lesions were significantly associated with AIFRS-specific survival rate. The present study confirmed that the presence of extrasinonasal LoCE reflecting a necrotic lesion, particularly at the skull base, was a poor independent prognostic factor of AIFRS-specific survival rate. In seven patients who expired because of the progression of AIFRS, extensive necrotic lesions of the body and/or greater wing of the sphenoid bone, orbital apex, and the adjacent large area dura were not completely resected. In our experience, fungal infections of soft tissue are sometimes treated with antifungal agents, whereas bone-infected fungi do not appear to be treated by antifungal agents alone. Postoperative endoscopic findings suggest that antifungal agents may be delivered from surrounding non-infected soft tissue to infected soft tissues because blood vessels and granulation tissue grow in adjacent healthy soft tissues, and some infected soft tissue can be removed at the clinic. However, infected bone may not be able to deliver antifungal agents because of thrombosis formation of penetrating vessels. The remaining lesions gradually spread to the adjacent area, eventually resulting in cerebrovascular injury and parenchymal damage. However, it is questionable as to whether surgery at these sites can be helpful for patients with AIFRS. These sites are adjacent to the cavernous sinus, internal carotid artery, and middle cranial fossa. Although focal necrotic lesions at these structures could be resected, extensive lesions could not be completely removed, and rather extensive resection might lead to serious morbidity, such as internal carotid artery injury, untreated CSF leakage, meningitis, or brain injury. Because fungi invading these structures also invaded paranasal sinuses, septum, turbinates, pterygopalatine fossa, and infratemporal fossa, a mucosal flap for skull base reconstruction, such as a nasoseptal flap, lateral nasal wall flap, and middle turbinate flaps, could not be used. These serious postoperative complications may significantly shorten the survival time.

Previous studies have shown that the involvement of brain, orbit, cavernous sinus, pterygopalatine fossa, and/or palate may lead to a poor prognosis^6,13,16–19^. However, our study showed that brain involvement is not associated with AIFRS-specific survival rate because four of six patients with intracranial fungal abscesses were successfully treated with antifungal agents and/or surgery. Furthermore, LoCE lesions in the orbit, cavernous sinus, infratemporal fossa, and meninges were significantly associated with AIFRS-specific survival rate in univariate analysis but not in multivariate analysis. A recent multicenter study also reported that orbit and intracranial involvement were not statistically significant prognostic factors^16^.

The present study included a small number of patients with AIFRS because of the rarity of this disease. Especially, the number of patients who died from AIFRS was small. Most of the patients could be successfully treated with surgical debridement combined with antifungal agents, probably because most of the suspected AIFRS patients were examined relatively quickly, underwent early MRI scans, and were operated upon when the extent of necrotic lesion was limited to a resectable area. In addition, this study was retrospective in design. However, based on our previous study^7^, we have identified a surgical strategy for AIFRS involving as complete as possible resection of LoCE lesions identified on preoperative Gd-enhanced MRI and additional resection of remaining or extended LoCE lesions identified on postoperative Gd-enhanced MRI. This consistent strategy may overcome the limitation of the small number of patients and retrospective design.

In conclusion, this study showed that patients with immunodeficiency who complain of facial pain, headache, and/or ophthalmoplegia should be suspected of having AIFRS. Although AIFRS has a relatively high mortality rate, complete resection of LoCE lesions identified on Gd-enhanced MRI can improve survival rates in patients with AIFRS. Under the provision of efficient antifungal agents and strict blood sugar control, the LoCE of the skull base was found to be an independent negative prognostic factor.

## Methods

### Study participants

In total, 69 patients were histologically diagnosed as having definite AIFRS and surgically treated between November 2002 and December 2018. All patients underwent surgery within 1 month of symptom onset (range, 1–29 days). Computed tomography (CT) and Gd-enhanced MRI were preoperatively performed. Endonasal endoscopic surgery was performed and procedures, including an additional sublabial approach, maxillectomy, orbital exenteration, and/or brain surgery, were performed as required. Postoperative MRI was performed to identify residual lesion and disease progression. An antifungal agent was administered before or after surgery according to the confirmation of the fungal species.

The extent of disease was defined as an enhanced lesion on Gd-enhanced MRI. To identify the specific extrasinonasal sites of disease associated with AIFRS-specific survival rate, nineteen patients whose disease was confined to the sinonasal cavity were excluded. The remaining 50 patients with preoperative disease beyond the sinonasal cavity, including the palate, orbit, skull base (bones structures including posterior table of frontal sinus, cribriform plate, ethmoid roof, and sphenoid bone that form the floors of the anterior, middle, and posterior cranial fossa), cavernous sinus, pterygopalatine fossa, infratemporal fossa, meninges, and brain, were included. Their clinical characteristics, preoperative and postoperative image findings, surgical findings, antifungal drug administration records, survival rate, and follow-up period were reviewed. The distribution of LoCE lesions identified on MRI were analyzed in the extrasinonasal areas.

### Ethics statement

This study was approved by the Institutional Review Board of the Asan Medical Center, which exempted the study from requiring individual patient consent. All procedures performed in studies were in accordance with the ethical standards of Institutional Review Board of the Asan Medical Center and with the 1964 Helsinki declaration and its later amendments or comparable ethical standards.

### Statistical analysis

The primary outcome of this study was disease-specific survival rate. The survival rates according to clinical factors were compared by using the log-rank test. Univariate and multivariate analyses of prognostic factors were performed by using Cox proportional hazards models. IBM^®^ SPSS^®^ Statistics for Windows, version 22.0 (IBM, Armonk, NY, USA) was used for all statistical analyses. Statistical differences were considered significant at *P* < 0.05.
